# Multiple imputation for analysis of incomplete data in distributed health data networks

**DOI:** 10.1038/s41467-020-19270-2

**Published:** 2020-10-29

**Authors:** Changgee Chang, Yi Deng, Xiaoqian Jiang, Qi Long

**Affiliations:** 1 University of Pennsylvania, Philadelphia, PA USA; 2 Emory University, Atlanta, GA USA; 3 University of Texas Health Science Center at Houston, Houston, TX USA

**Keywords:** Computational science, Statistics

## Abstract

Distributed health data networks (DHDNs) leverage data from multiple sources or sites such as electronic health records (EHRs) from multiple healthcare systems and have drawn increasing interests in recent years, as they do not require sharing of subject-level data and hence lower the hurdles for collaboration between institutions considerably. However, DHDNs face a number of challenges in data analysis, particularly in the presence of missing data. The current state-of-the-art methods for handling incomplete data require pooling data into a central repository before analysis, which is not feasible in DHDNs. In this paper, we address the missing data problem in distributed environments such as DHDNs that has not been investigated previously. We develop communication-efficient distributed multiple imputation methods for incomplete data that are horizontally partitioned. Since subject-level data are not shared or transferred outside of each site in the proposed methods, they enhance protection of patient privacy and have the potential to strengthen public trust in analysis of sensitive health data. We investigate, through extensive simulation studies, the performance of these methods. Our methods are applied to the analysis of an acute stroke dataset collected from multiple hospitals, mimicking a DHDN where health data are horizontally partitioned across hospitals and subject-level data cannot be shared or sent to a central data repository.

## Abstract (web summary)

Distributed health data networks (DHDNs) leverage data from multiple healthcare systems, but often face major analytical challenges in the presence of missing data. This paper develops distributed multiple imputation methods that do not require sharing subject-level data across health systems.

## Introduction

In the past two decades, enormous amounts of health data have been collected and digitized, partly due to increasingly broader adoption of electronic health records (EHRs) by many healthcare systems. Pooling such big health data from multiple institutions such as healthcare systems and health insurance companies into a single database increases sample sizes for subsequent data analyses and, more importantly, the pooled data can provide a more representative sample of a larger population of interest. As such, it offers great promises in improving the validity, robustness and generalizability of research findings. However, pooling data from multiple institutions may not always be feasible or desirable. First, when the amount of data is massive and continues to grow, it may not be feasible or efficient to transmit data between institutions or store all data in one central repository. Second, for big health data, it may be desirable to store them in a distributed fashion and take advantage of advances in parallel computing. Third, most importantly, due to government regulations, institutional policies, and privacy concerns, it may not be possible to transfer big health data at the patient level from one institution to another or there are extremely high hurdles for such data transferring that may take years to clear. For example, Veteran’s Health Administration policies require its EHR data to remain only within VA’s facilities. In addition, improper disclosure of individual-level data has serious implications, such as discrimination for employment, insurance, or education^1^. In addition, the current standard practice of data de-identification through removing indiviual identifiers is inadequate for privacy protection in the era of big data, as a large body of research has demonstrated that given some background information of an individual, an adversary can learn (from “de-identified” data) sensitive information about the victim^2–6^.

To address these challenges, distributed health data networks (DHDNs) that can store and analyze EHRs data from multiple sites without sharing individual-level data have drawn increasing interests in recent years^7,8^. Examples of DHDNs^9^, include the vaccine safety datalink, the health care systems research network, the sentinel initiative, and most recently the patient-centered SCAlable national network for effectiveness research (pSCANNER)^10^ that is part of PCORnet. To enhance scalability and privacy protection in distributed analysis, the PopMedNet platform^11,12^ has been developed to provide software enabled governance over shared data. DHDNs eliminate the need to create, maintain, and secure access to central data repositories, minimize the need to disclose protected health information outside the data-owning entity, and mitigate many security, proprietary, legal, and privacy concerns. In this work, we focus on horizontally partitioned data^13^, meaning that different data custodians such as hospitals and healthcare providers have the same set of features for different sets of patients. For example, several healthcare systems are interested in analyzing pooled data from their EHRs to improve the precision and generalizbility of analysis results. However, due to the aforementioned concerns, they are not allowed or are reluctant to share individual-level data with others, despite the substantial benefits from such collaboration. DHDNs would lower the hurdles for them to collaborate in a distributed analysis environment^14^, highlighted needed methods contributions to analysis of distributed EHRs data.

As EHRs are collected as part of healthcare delivery, missing data are pervasive in EHRs and DHDNs^8,15^. Missing data problem reduces the usable sample size and hence analysis power. Improper handling of missing data is known to compromise the validity of analysis and yield biased results, and could subsequently lead to inappropriate healthcare and health policy decisions. To choose the best way forward in handling missing data, the pattern and mechanism of missingness need to be considered^16^. Three main missing data mechanisms are missing completely at random (MCAR), missing at random (MAR), and missing not at random^17^. Most of the existing methods for handling missing data rely on the assumption of MAR, i.e., missingness only depends on observed data, and which is the focuse of our current work as well.

Multiple imputation (MI)^17^ is arguably the most popular method for handling missing data largely due to its ease of use. MI methods replace each missing value with samples from its posterior predictive distribution. The predictive imputation model is estimated from the observed data, which have no missing values. The missing values are imputed multiple times in order to account for the the uncertainty of imputation, and then each imputed dataset is used to fit the analysis model parameters ***θ***^18^ proposed a simple method for combining these analysis results from multiple imputed datasets, which is known as Rubin’s rule. In the presence of general missing data patterns, the MI by chained equations (MICE) method is widely adopted and has been shown to achieve superior performance in practice^19,20^.

While there has been a large body of literature on handling missing data, there has been little work on handling distributed incomplete data such as missing data in DHDNs. Of note, while pSCANNER^10^ has developed a suit of software tools for privacy-preserving distributed data analysis, it currently has no tools for handling distributed missing data.

To enhance protection of patient privacy, we investigate distributed MI methods for handling missing data that do not require sharing individual level data between sites. Under MAR, one straightforward privacy-preserving MI approach for horizontally partitioned incomplete data would be to conduct MI within each institution/site and then perform the distributed analysis. We call this approach the independent MI (iMI). The iMI has a number of limitations. In particular, it fails to leverage data from other sites, which leads to large variability in imputation and loss of power in subsequent analysis. This becomes more pronounced as the proportion of missing data in individual sites increases. In the extreme case when one variable is missing for all observations in a single site, this variable cannot be imputed in that site using the iMI approach. As a result, the data from this site may not be used in any subsequent analysis where that variable is needed^21^ proposed a privacy-preserving lazy decision-tree imputation algorithm for data that are horizontally partitioned between two sources. As their algorithm is designed for only single imputation, it is challenging to conduct proper statistical inference such as hypothesis testing using their singly imputed dataset that underestimates the uncertainty of imputation. In addition, it is not directly applicable to general missing data patterns and the case of more than two sources, and their complex decision tree algorithm may overfit the data and may not be communication efficient.

Since communicating data between sites in distributed learning can be a costly operation, we seek to develop communication-efficient distributed MI approaches. The aforementioned naïve iMI approach is communication-efficient as it involves no communication between sites. We propose two additional communication-efficient approaches, inspired by the inference methods for distributed complete data (CD); the average mixture approach (AVGM) and the communication-efficient surrogate likelihood (CSL) approach. In the AVGM approach^22^, each site finds the local estimate using the data available at the site, and then these estimates are averaged to find the global estimate. The CSL approach^23^ uses the curvature information from a central site and the pooled derivative at a point near the true parameter. AVGM is expected to perform better when the samples are evenly distributed across the sites, while CSL is expected to perform well when the central site has the majority of samples. We will use these two approaches to develop distributed MI approaches, avgmMI and cslMI, for univariate missing data patterns. In addition, we develop the another distributed MI method that uses only the aggregated statistics from each site that are sufficient to obtain the same global estimate as if one had access to data pooled from all sites, and we call this method siMI. siMI can be communication efficient for linear regression models but not for nonlinear regression models for which the fitting algorithm is iterative. Of note, similar to iMI, when one variable is missing for all observations in a single site, this variable cannot be imputed in that site using avgmMI and cslMI and hence the data from this site may not be used in any subsequent analysis where that variable is needed. However, siMI would enable the use of data from these sites, which is one advantage of siMI over the other methods. Using these techniques, we also develop distributed MICE methods for general missing data patterns. However, since the standard MICE algorithm involves fitting of the imputation model multiple times, these direct extensions may not be as communication efficient except for iMICE which requires no communication.

Our work represents the first attempt to develop MI methods that allow proper statistical inference such as hypothesis testing in analysis of horizontally partitioned incomplete data in DHDNs. The remainder of the article is organized as follows. In the section “Results”, we assess the strengths and weaknesses of the proposed distributed imputation methods in simulation studies; we then apply the methods to analysis of an acute stroke dataset collected from EHRs of multiple hospitals, mimicking a DHDN setting. The section “Discussion” provides some concluding remarks. In the section “Methods”, we first briefly review the standard MI and the two distributed analysis methods for CD, AVGM, and CSL, respectively, and then present our communication-efficient distributed MI and MICE methods, respectively.

## Results

### Simulation studies

We conduct simulation studies to investigate strengths and limitations of the four privacy-preserving distributed MI methods described in the section “Methods” under the MAR assumption. We consider a linear regression model as the “analysis model”

1 $${\bf{y}}={\theta }_{0}+{\theta }_{1}{{\bf{x}}}_{1}+\cdots +{\theta }_{p}{{\bf{x}}}_{p}+{\boldsymbol{\epsilon }},$$

where **y** is the *N* × 1 vector of responses *Y*, **x**_1_, …, **x**_*p*_ are the *N* × 1 covariate vectors for variables *X*_1_ through *X*_*p*_, ***θ*** = (*θ*_0_, *θ*_1_, …, *θ*_*p*_) denotes the model parameters of interest, and ${\boldsymbol{\epsilon }} \sim {\mathcal{N}}(0,{\sigma }^{2}{\bf{I}})$ is the *N* × 1 vector of errors. We investigate both univariate and general missing data patterns. We apply each distributed MI method to the simulated missing data and then fit the analysis model using the imputed data to evaluate the imputation performance in terms of bias and SD of regression coefficient estimates, and communication costs. To benchmark the performance of the distributed MI methods, we compare their results with the results from the CD analysis which fits the analysis model using the full data before missing values are generated, and the results from the complete case analysis which fits the analysis model using only the set of complete cases that have all variables observed after missing values are generated.

In the first scenario, we have two continuous variables (*p* = 2). The first variable *X*_1_ has missing values while *X*_2_ is fully observed. For each subject, *X*_2_ is first generated from a uniform distribution ${\mathcal{U}}(-3,3)$. Given *X*_2_, variable *X*_1_ is sampled from a normal distribution with mean *μ*_*X*_ = 0.2 − 0.5*X*_2_ and variance ${\sigma }_{X}^{2}=1$. The outcome *Y* is generated from *Y* = *θ*_0_ + *θ*_1_*X*_1_ + *θ*_2_*X*_2_ + *ϵ*, where $\epsilon \sim {\mathcal{N}}(0,1)$ and all *θ*_*j*_ = 1(*j* = 0, 1, 2). Variable *X*_1_ is missing with probability ${\{1+\exp (-0.3+0.2Y-0.1{X}_{2})\}}^{-1}$, resulting in approximately 50% of missing rate.

In the second scenario, we make only one change from the first scenario, which is *X*_1_ is now a binary variable. Given *X*_2_, instead of sampling *X*_1_ from a normal distribution, we generate *X*_1_ from a Bernoulli distribution ${\mathcal{B}}(1,p)$ with probability $p={\{1+\exp (-0.2+0.5{X}_{2})\}}^{-1}$. The outcome variable *Y* and the missingness of *X*_1_ are generated in the same way as in the first scenario. The resulting missing rate is about 50%.

The third scenario considers general missing data patterns. We have *p* = 5 predictor variables, and *X*_1_–*X*_3_ have missing values. The fully observed variables *X*_4_ and *X*_5_ are independent and identically distributed as ${\mathcal{N}}(0,1)$. Given *X*_4_ and *X*_5_, we sample *X*_1_–*X*_3_ from a multivariate normal distribution ${\mathcal{N}}({{\boldsymbol{\mu }}}_{X},{\Sigma }_{X})$ with

$${{\boldsymbol{\mu }}}_{X}=(0.3-0.3{X}_{4}-0.1{X}_{5}){\bf{1}},\quad {\Sigma }_{X}=\left[\begin{array}{lll}1&0.5&0.5\\ 0.5&1&0.5\\ 0.5&0.5&1\end{array}\right].$$

The outcome *Y* is generated by *Y* = *θ*_0_ + *θ*_1_*X*_1_ + *θ*_2_*X*_2_ + *θ*_3_*X*_3_ + *θ*_4_*X*_4_ + *θ*_5_*X*_5_ + *ϵ*, where $\epsilon \sim {\mathcal{N}}(0,1)$ and all *θ*_*j*_ = 1(*j* = 0, 1, …, 5). Missing values in *X*_1_–*X*_3_ are generated based on the logistic regression models for the missing indicators *δ*_1_–*δ*_3_.

$${\rm{logit}}(\Pr ({\delta }_{1}=1))=-1.0-0.4Y-0.1{X}_{4}-0.2{X}_{5},\\ {\rm{logit}}(\Pr ({\delta }_{2}=1))=-0.8-0.6Y+0.2{X}_{4}+0.4{X}_{5},\\ {\rm{logit}}(\Pr ({\delta }_{3}=1))=-0.8-1.0Y+0.4{X}_{4}+0.3{X}_{5},$$

resulting in 20% of missing rates for each missing variable and 50% of complete case rate.

Let *K* be the number of data sites distributed over the network. We consider two different numbers of sites (*K* = 5, 10) and three different sample sizes *N* = 250, 500, and 1000. We also look at two different types of distributions among the samples over the sites. In the first type (U), the samples are unevenly distributed. The first site has the majority of the samples and each site except the first has 15 samples only. In the second type (E), the samples are evenly distributed over the *K* sites. Table 1 lists all 15 settings of *K*, *N*, and the sample distribution type which are considered in this study. To evaluate the performance, we compute bias, standard deviation (SD), and root mean squared error of the estimates for ***θ*** from 1000 Monte Carlo datasets, which are defined as ${\rm{Bias}}({\boldsymbol{\theta }})=\, \parallel {\mathbb{E}}{\boldsymbol{\theta }}-{{\boldsymbol{\theta }}}_{0}{\parallel }_{2}$, ${\rm{SD}}({\boldsymbol{\theta }})=\sqrt{{\mathbb{E}}\parallel {\boldsymbol{\theta }}-{\mathbb{E}}{\boldsymbol{\theta }}{\parallel }_{2}^{2}}$, and ${\rm{rMSE}}({\boldsymbol{\theta }})=\sqrt{{\mathbb{E}}\parallel {\boldsymbol{\theta }}-{{\boldsymbol{\theta }}}_{0}{\parallel }_{2}^{2}}$, where ***θ***_0_ is the true value of ***θ***.

**Table 1:** Fifteen different distributions of samples.

Type	*K*	*N*	*n*^(1)^	*n*^(2)^	*n*^(3)^	*n*^(4)^	*n*^(5)^	*n*^(6)^	*n*^(7)^	*n*^(8)^	*n*^(9)^	*n*^(10)^
–	1	250	250									
–	1	500	500									
–	1	1000	1000									
U	5	250	190	15	15	15	15					
U	5	500	440	15	15	15	15					
U	5	1000	940	15	15	15	15					
U	10	250	115	15	15	15	15	15	15	15	15	15
U	10	500	365	15	15	15	15	15	15	15	15	15
U	10	1000	865	15	15	15	15	15	15	15	15	15
E	5	250	50	50	50	50	50					
E	5	500	100	100	100	100	100					
E	5	1000	200	200	200	200	200					
E	10	250	25	25	25	25	25	25	25	25	25	25
E	10	500	50	50	50	50	50	50	50	50	50	50
E	10	1000	100	100	100	100	100	100	100	100	100	100

Type indicates whether the samples are unevenly (U) distributed or evenly (E) distributed. *K* is the number of sites. *N* is the total number of samples.

Tables 2–4 summarize the results for scenarios 1–3, respectively. Note that the CC method is biased regardless of the sample size *N*, which is as expected since the missing mechanism is not MCAR. Overall, the biases of all MI methods deteriorate as *N* decreases and *K* increases. However, the changes vary with the method, the type of sample distribution, and the type of imputed variable.

**Table 2:** Simulation results for scenario 1 where a continuous variable *X*_1_ has missing values.

			*N* = 250				*N* = 500				*N* = 1000			
Type	*K*	Method	Bias	SD	rMSE	Com	Bias	SD	rMSE	Com	Bias	SD	rMSE	Com
–	1	CD	0.001	0.103	0.103	0.0	0.001	0.074	0.074	0.0	0.001	0.052	0.052	0.0
		CC	0.104	0.153	0.185	0.0	0.105	0.107	0.150	0.0	0.106	0.075	0.130	0.0
U	5	iMI	0.169	0.224	0.281	0.0	0.105	0.178	0.206	0.0	0.062	0.132	0.146	0.0
		avgmMI	0.022	0.136	0.138	2.0	0.012	0.093	0.094	2.0	0.004	0.065	0.065	2.0
		cslMI	0.002	0.133	0.133	3.0	0.001	0.093	0.093	3.0	0.003	0.065	0.065	3.0
		siMI	0.002	0.131	0.131	2.0	0.002	0.093	0.093	2.0	0.003	0.064	0.064	2.0
	10	iMI	0.325	0.256	0.414	0.0	0.208	0.215	0.299	0.0	0.133	0.177	0.221	0.0
		avgmMI	0.054	0.140	0.150	2.0	0.029	0.094	0.098	2.0	0.013	0.065	0.067	2.0
		cslMI	0.003	0.138	0.138	3.0	0.002	0.093	0.093	3.0	0.003	0.065	0.065	3.0
		siMI	0.003	0.130	0.130	2.0	0.002	0.092	0.092	2.0	0.004	0.065	0.065	2.0
E	5	iMI	0.068	0.131	0.147	0.0	0.033	0.092	0.098	0.0	0.018	0.064	0.067	0.0
		avgmMI	0.026	0.133	0.135	2.0	0.011	0.092	0.093	2.0	0.004	0.065	0.065	2.0
		cslMI	0.021	0.170	0.171	3.0	0.003	0.111	0.111	3.0	0.002	0.076	0.076	3.0
		siMI	0.004	0.130	0.130	2.0	0.003	0.091	0.091	2.0	0.004	0.065	0.065	2.0
	10	iMI	0.180	0.140	0.228	0.0	0.076	0.092	0.119	0.0	0.038	0.065	0.075	0.0
		avgmMI	0.063	0.136	0.150	2.0	0.031	0.093	0.098	2.0	0.013	0.065	0.066	2.0
		cslMI	0.150	0.348	0.378	3.0	0.024	0.154	0.156	3.0	0.003	0.091	0.091	3.0
		siMI	0.004	0.130	0.130	2.0	0.003	0.092	0.092	2.0	0.004	0.064	0.065	2.0

Reported are Bias, average bias; SD, Monte Carlo standard deviation; rMSE, root mean squared error; Com, number of communications. *N* is the total number of samples. See Table 1 for local sample sizes. Results are based on 1000 Monte Carlo datasets.

**Table 3:** Simulation results for scenario 2 where a binary variable *X*_1_ has missing values.

			*N* = 250				*N* = 500				*N* = 1000			
Type	*K*	Method	Bias	SD	rMSE	Com	Bias	SD	rMSE	Com	Bias	SD	rMSE	Com
–	1	CD	0.013	0.348	0.349	0.0	0.004	0.239	0.239	0.0	0.004	0.166	0.166	0.0
		CC	0.402	0.491	0.635	0.0	0.408	0.347	0.535	0.0	0.407	0.239	0.472	0.0
U	5	iMI	0.077	0.444	0.451	0.0	0.046	0.324	0.327	0.0	0.023	0.227	0.229	0.0
		avgmMI	0.052	0.585	0.587	2.0	0.059	0.441	0.445	2.0	0.056	0.293	0.298	2.0
		cslMI	0.014	0.472	0.472	3.0	0.005	0.332	0.332	3.0	0.004	0.226	0.226	3.0
		siMI	0.014	0.468	0.468	10.7	0.005	0.331	0.331	10.2	0.003	0.232	0.232	9.7
	10	iMI	0.180	0.404	0.442	0.0	0.105	0.309	0.326	0.0	0.051	0.222	0.228	0.0
		avgmMI	0.115	0.624	0.635	2.0	0.128	0.502	0.518	2.0	0.119	0.333	0.354	2.0
		cslMI	0.014	0.470	0.470	3.0	0.005	0.331	0.331	3.0	0.003	0.230	0.231	3.0
		siMI	0.014	0.469	0.469	10.7	0.005	0.329	0.329	10.2	0.003	0.229	0.229	9.7
E	5	iMI	0.056	0.447	0.451	0.0	0.037	0.324	0.327	0.0	0.019	0.229	0.230	0.0
		avgmMI	0.108	0.524	0.535	2.0	0.047	0.348	0.351	2.0	0.022	0.233	0.234	2.0
		cslMI	0.016	0.533	0.533	3.0	0.005	0.344	0.344	3.0	0.004	0.233	0.233	3.0
		siMI	0.014	0.469	0.469	10.7	0.004	0.330	0.330	10.2	0.002	0.229	0.229	9.7
	10	iMI	0.169	0.413	0.446	0.0	0.081	0.319	0.329	0.0	0.041	0.226	0.230	0.0
		avgmMI	0.226	0.653	0.691	2.0	0.125	0.386	0.406	2.0	0.054	0.241	0.247	2.0
		cslMI	0.030	0.792	0.793	3.0	0.007	0.446	0.446	3.0	0.003	0.259	0.259	3.0
		siMI	0.015	0.467	0.467	10.7	0.004	0.330	0.330	10.2	0.003	0.228	0.228	9.7

Reported are Bias, average bias; SD, Monte Carlo standard deviation; rMSE, root mean squared error; Com, number of communications. *N* is the total number of samples. See Table 1 for local sample sizes. Results are based on 1000 Monte Carlo datasets.

**Table 4:** Simulation results for scenario 3 where three continuous variables *X*_1_–*X*_3_ have missing values.

			*N* = 250				*N* = 500				*N* = 1000			
Type	*K*	Method	Bias	SD	rMSE	Com	Bias	SD	rMSE	Com	Bias	SD	rMSE	Com
–	1	CD	0.004	0.179	0.179	0	0.004	0.127	0.127	0	0.004	0.089	0.089	0
		CC	0.363	0.240	0.435	0	0.365	0.167	0.401	0	0.365	0.117	0.383	0
U	5	iMICE	0.067	0.258	0.267	0	0.037	0.176	0.180	0	0.022	0.121	0.123	0
		avgmMICE	0.011	0.218	0.218	1290	0.006	0.151	0.151	1290	0.004	0.106	0.106	1290
		cslMICE	0.005	0.214	0.214	1935	0.004	0.151	0.151	1935	0.004	0.106	0.106	1935
		siMICE	0.004	0.213	0.213	1290	0.004	0.150	0.150	1290	0.004	0.106	0.106	1290
	10	iMICE	0.146	0.286	0.321	0	0.080	0.201	0.216	0	0.047	0.138	0.146	0
		avgmMICE	0.022	0.221	0.222	1290	0.011	0.153	0.154	1290	0.006	0.106	0.106	1290
		cslMICE	0.005	0.215	0.215	1935	0.004	0.150	0.150	1935	0.005	0.106	0.106	1935
		siMICE	0.004	0.213	0.213	1290	0.004	0.150	0.150	1290	0.005	0.105	0.105	1290
E	5	iMICE	0.027	0.217	0.219	0	0.014	0.151	0.152	0	0.009	0.105	0.106	0
		avgmMICE	0.013	0.215	0.215	1290	0.006	0.151	0.151	1290	0.004	0.105	0.105	1290
		cslMICE	0.019	0.243	0.244	1935	0.006	0.156	0.156	1935	0.005	0.107	0.108	1935
		siMICE	0.005	0.213	0.213	1290	0.005	0.150	0.150	1290	0.004	0.105	0.105	1290
	10	iMICE	0.076	0.228	0.240	0	0.032	0.152	0.155	0	0.016	0.106	0.108	0
		avgmMICE	0.024	0.219	0.221	1290	0.012	0.151	0.152	1290	0.006	0.106	0.106	1290
		cslMICE	–	–	–	–	0.020	0.194	0.195	1935	0.006	0.114	0.114	1935
		siMICE	0.005	0.213	0.213	1290	0.004	0.150	0.150	1290	0.004	0.105	0.105	1290

Reported are Bias, average bias; SD, Monte Carlo standard deviation; rMSE, root mean squared error; Com, number of communications. The cslMICE method failed in a few cases due to instability when *N* = 250 samples are evenly (E) distributed over *K* = 10 sites. *N* is the total number of samples. See Table 1 for local sample sizes. Results are based on 1000 Monte Carlo datasets.

We can see that iMI and iMICE are less biased when the samples are evenly distributed, as each individual imputation model can be fitted stably. In contrast, when most of sites do not have enough samples, the individual and hence the aggregated estimates are less stable. Note that avgmMI and avgmMICE are hardly affected by the type of sample distribution when the missing variable is continuous (scenarios 1 and 3). However, they are substantially influenced when the missing variable is binary (scenario 2). The difference is even bigger when *K* = 10. Conversely, cslMI and cslMICE are worse when the samples are evenly distributed, obviously because the sample size at the central site is smaller. Note that they utilize the curvature information from the central site only and the initial estimate is obtained from the central site as well. Therefore, its performance is sensitive to the sample size at the central site. In particular, note that a few cases of cslMICE failed to converge in evenly distributed case when *K* = 10 and *N* = 250. However, the performance of the CSL based methods is comparable to that of siMI and siMICE when the central site has the majority of the samples. Note that the estimates of siMI and siMICE are as unbiased as comparable to CD in all settings, although they suffer a bit larger SDs.

Note that iMI and iMICE do not require any communication for imputation. The avgmMI approach only requires two one-way communications; one to fit the imputation model by AVGM and another to deliver the aggregated estimates to all sites for imputation. The cslMI approach requires one more one-way communication; two to fit the imputation model by CSL and another to deliver the estimated estimates to all sites for imputation. However, the cslMI method transmits vectors only in the first two communications, while avgmMI sends an estimate vector and a covariance matrix in every communication. The siMI approach requires as many communications as avgmMI does when the imputation model is a linear regression. However, siMI requires more communications when the imputation model is nonlinear as shown in Table 3.

As we can see in Table 4, the communication costs of the proposed MICE methods are huge except iMICE. Due to the iterative nature of the MI by chained equation, the number of required communications is proportional to the number of imputations, *M*.

### Analysis of real data

The Georgia Coverdell Acute Stroke Registry (GCASR), covering nearly 80% of acute stroke admissions in the state of Georgia in USA, was set up to monitor and improve the care of acute stroke patients in the prehospital and hospital settings. The GCASR dataset analyzed in this section includes 68,287 patients from 75 hospitals in Georgia with clinically diagnosed acute stroke between 2005 and 2013. The data collected from EHRs in each hospital include a total of 203 variables, many of which have missing values due to various reasons. The goal of our analysis is to fit a linear regression model for assessing the effect of 14 features on the outcome variable of arrival-to-computed tomography time, an important quality indicator for acute stroke care, in the presence of missing data. The features of interest include patient-related characteristics such as age and gender, and pre-hospital-related characteristics such as EMS notification.

To assess the performance of the distributed imputation methods, we consider the case where EHRs data from individual hospitals cannot be pooled or sent to a central data repository, mimicking a DHDN, and we seek to impute missing values in this distributed set-up while protecting data privacy. Among these features of interest, only gender and race are observed for all patients, and the missing rates for the other variables range from 0.04 to 50.73%. Of note, in some hospitals one or more variables (e.g., NIH stroke score, EMS prenotification, and NPO) are missing for all observations. Since iMICE cannot be used to impute missing values in a hospital with one or more variables missing for all observations, such hospitals were removed in the first set of analyses resulting in 67,944 observations from 66 hospitals. The sample size in each hospital ranges from 18 to 4,333 with median 578. The number of complete cases across all hospitals is 13,353.

As with the simulation study, we used the CC, iMICE, avgmMICE, cslMICE, and siMICE methods. The CD analysis is not applicable to real data analysis. In addition, since the cslMICE approach is sensitive to the sample size of the central site, we consider two versions of cslMICE, namely, cslMICE(M) and cslMICE(m). For cslMICE(M), the central site is chosen to be the one with the most samples (4333). For cslMICE(m), the central site is the hospital with the median sample size (578). For each imputation method, we generate *M* = 20 imputed datasets. To benchmark the performance of the distributed MI methods, we also include the results from the complete case analysis, noting that the CD analysis is not applicable in the real data example.

To compare the performance of distributed imputation methods without being complicated by the choice of distributed method for fitting the analysis model, we chose to fit the analysis model using the imputed data pooled across all hospitals. The analysis results from the *M* = 20 imputed datasets are combined using the Rubin’s rule. Specifically, let ${\widehat{{\boldsymbol{\theta }}}}_{m}$ and $\widehat{{\rm{Var}}}({\widehat{{\boldsymbol{\theta }}}}_{m})$ be the regression coefficient estimate and its estimated variance (or variance–covariance) from the *m*-th imputed dataset. Then the overall coefficient estimate is given by $\widehat{{\boldsymbol{\theta }}}=\frac{1}{M}{\sum }_{m}{\widehat{{\boldsymbol{\theta }}}}_{m}$, and its estimated variance is given by $\widehat{{\rm{Var}}}(\widehat{{\boldsymbol{\theta }}})=\frac{1}{M}{\sum }_{m}\widehat{{\rm{Var}}}({\widehat{{\boldsymbol{\theta }}}}_{m})+\frac{1}{M-1}{\sum }_{m}({\widehat{{\boldsymbol{\theta }}}}_{m}-\widehat{{\boldsymbol{\theta }}}){({\widehat{{\boldsymbol{\theta }}}}_{m}-\widehat{{\boldsymbol{\theta }}})}^{T}$.

**Fig. 1:**
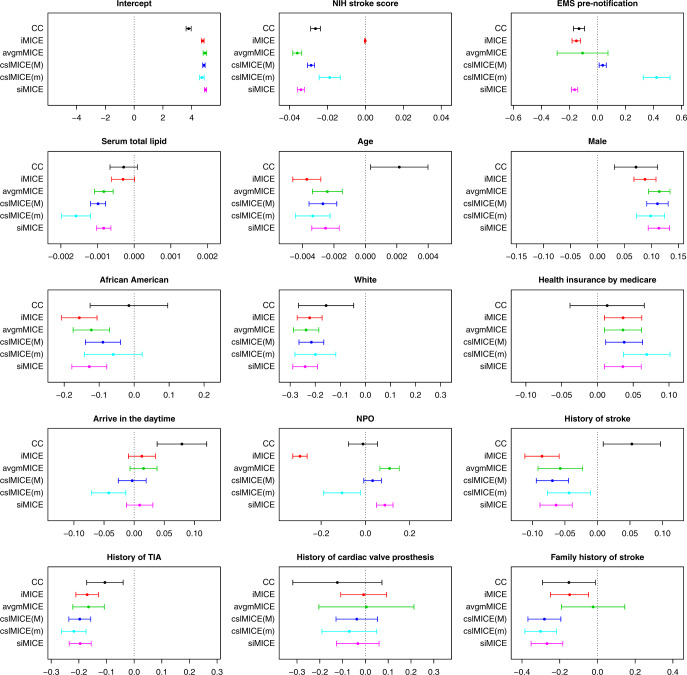
Forest plot for analysis results of the GCASR data. The parameter estimates (dots) and associated 95% confidence intervals (whiskers) for each regression coefficient including the intercept are compared between all the methods. The hospitals in which at least one variable is missing for all observations have been removed for iMICE. The plots are based on 67,944 observations from 66 hospitals. The sample size in each hospital ranges from 18 to 4333 with median 578.

Figure 1 presents the parameter estimates and associated 95% confidence intervals for each regression coefficient in the linear regression model of interest. Of note, the hospitals in which at least one variable is missing for all observations are removed for iMICE since the missing values in such hospitals cannot be imputed using iMICE. For each method other than siMICE, we counted the number of discrepancies in statistical significance defined at *α* = 0.05 or in sign/direction of estimated effect compared to siMICE. Table 5 reports the number of discrepancies along with the number of communications required for each imputation method.

**Table 5:** Comparisons of analysis results of the GCASR data.

	CC	iMICE	avgmMICE	cslMICE(M)	cslMICE(m)	siMICE
# of communications	0	0	4730	7095	7095	25,397
# of discrepancies	8	3	2	2	4	–

Reported are the communication costs and the number of discrepancies in statistical significance defined at *α* = 0.05 or in sign/direction of estimated effect compared against siMICE. The hospitals in which at least one variable is missing for all observations are removed for iMICE.

Since the results from the siMICE method are the same as the results from the standard MICE using pooled data, the latter is omitted from Fig. 1, and the results from the siMICE method are used to benchmark the other methods. As shown in Table 5, siMICE incurs substantially higher communication costs than the other distributed MI methods. Compared to the results from siMICE, the CC analysis yields substantially different parameter estimates for multiple regression coefficients as well as disagreement in statistical significance or in direction of estimated effect for eight features. This demonstrates the need for adequate handling missing data in the analysis of the GCASR data.

The results from the other distributed MI methods are closer to the results from siMICE than the CC analysis. Though, cslMICE(m), which uses the hospital with the medium sample size as the central site, exhibits the second largest number of discrepancies after CC, including disagreement for four features. While offering substantial savings in communication costs and yielding similar parameter estimates for some features, iMICE shows notable discrepancies for “NIH stroke score,” “Serum total lipid”, and “NPO” when compared to siMICE. On the other hand, cslMICE(M), which uses the hospital with the largest sample size as the central site, and avgmMICE yield the smallest number of discrepancies from siMICE, specifically only two discrepancies as shown in Table 5. In addition, Fig. 1 provides more granular information about comparing cslMICE(M) and avgmMICE with siMICE. The locations of 95% confidence intervals obtained by avgmMICE are most similar to those obtained by siMICE, with the only notable discrepancy for “family history of stroke.” The lengths of confidence intervals obtained by cslMICE(M) are most similar to those obtained by siMICE, which could be attributed to the fact that it uses the curvature information of the central site with a large sample size. As a comparison, avgmMICE yields wider intervals for a number of features including “EMS prenotification,” “history of TIA,” “history of cardiac valve prosthesis,” and “family history of stroke.”

To assess the performance of the distributed MI methods when sample sizes in individual hospitals are moderate to small, we conducted another set of analyses after removing the hospitals with more than *T* patients where *T* = 500, 300, 100. When *T* = 500, the total sample size decreases to 5307 patients from 26 hospitals. The number of patients in each hospital ranges from 18 to 462 with a median of 163 and the number of complete cases is only 362. In this set of analyses, we exclude cslMICE(m). As *T* decreases and, in other words, the number of patients per hospital continues to decrease, the discrepancies between the results from siMICE and the results from the other distributed MI methods including iMICE, avgmMICE and cslMICE(M) become greater. Particularly, the discrepancies between siMICE and cslMICE(M) tend to grow faster than the discrepancies between siMICE and avgmMICE, suggesting that cslMICE(M) is more sensitive to moderate to small sample sizes in all sites than avgmMICE.

## Discussion

In this paper, we consider the problem of distributed incomplete data where data from multiple sites are not allowed to be combined, due to institutional policies or privacy concerns. We have developed and investigated four MI approaches that allow proper statistical inference such as hypothesis testing in analysis of horizontally partitioned incomplete data in DHDNs.

Our numerical experiments provide insights into the strengths and weaknesses of these methods. The proposed distributed imputation methods except for iMI/iMICE enable the use of data from all sites including sites with one or more variables missing for all observations. siMI has been shown in our numerical studies to yield comparable performance as the standard MI using pooled data, but it is not communication efficient for generalized linear imputation models. While cslMI and avgmMI are more communication efficient for all imputation models, their performance may be sensitive to sample sizes in individual sites. In particular, the performance of cslMI may become unstable as the sample size in the central site becomes small to moderate. While avgmMI is less sensitive to small sample sizes, our simulations show that it tends to yield larger bias when imputing binary variables. Of note, siMI may be particularly appealing when analyzing data for uncommon diseases for which the sample size can be small in each individual site and missing data can further complicate data analysis. On the other hand, given that existing networks have 5 to upwards of 70 or more sites and can have millions to billions of records, the avgmMI and cslMI approaches can be very appealing options compared to the iMI or siMI approaches if all the data are used in analysis.

Unlike the other proposed methods, iMI requires no communication between sites but may lead to unstable results as the sample sizes in some sites become small to moderate. As shown in the real data example, when one variable, say *X*_1_, is missing for all observations in a single site, *X*_1_ in that site cannot be imputed using iMI/iMICE and hence the data from the site may not be used in subsequent analysis involving *X*_1_. The choice of distributed imputation approaches may also depend on whether data heterogeneity across multiple sites can be adequately adjusted for in imputation models. If we are able to adequately account for the heterogeneity by say including covariates that capture the heterogeneity or random effects for sites in imputation models, the siMI, cslMI, and avgmMI methods that borrow information across sites can enhance the efficiency of imputation and hence the power of subsequent analysis of imputed datasets. However, if that is not the case, then the iMI approach may be preferred.

We have investigated the extensions of the distributed MI methods for general missing patterns through the use of chained equations (MICE). Although these methods are privacy-preserving and yield good performance, they are not communication efficient as demonstrated in the numerical experiments. In cases where communication costs are of critical concern, more communication-efficient imputation methods are needed for handling general missing data as potential future work. Another potential limitation is that siMI, cslMI and avgmMI may not always be privacy-preserving as the summary statistics transmitted between individual sites and a central server may still leak individual-level information^24^. Particularly, the siMI method needs to transfer the entire design matrix between sites, which poses higher risk of leaking individual patients’ information. To address this issue, a differential privacy step^25^ can be added to further strengthen the privacy-preserving property.

In practice, robust imputation methods such as predictive mean matching (PMM) and random forest (RF) imputation are widely used. It is of future interest to develop distributed versions of generic imputation methods include PMM and RF, which, however, can be very challenging. Such distributed generic imputation methods are expected to require additional communication overhead and more general definition of sufficient statistics. Particularly, the information to be exchanged for a distributed generic imputation method needs to be carefully investigated, while taking into account statistical validity, privacy-preserving property, and communication costs.

## Methods

### Ethical approval

This study was reviewed by the Institutional Review Board at the University of Pennsylvania which determined that the study does not meet the criteria for human subject research since it involves only secondary analysis of de-identified data from an existing database and does not involve new data collection.

### Notation

To fix ideas, suppose that we are interested in fitting the analysis model (1) of outcome *Y* on *p* features *X*_1_, …, *X*_*p*_, using a random sample of *N* observations. We define **x**_0_ = **1** for the *N* × 1 vector of ones and denote the values for the *i*-th individual by ${\widetilde{{\bf{x}}}}_{i}={({x}_{i0} = 1,{x}_{i1},\ldots ,{x}_{ip})}^{T}$ and *y*_*i*_. Let ${\bf{X}}=[{\bf{1}}\ {{\bf{x}}}_{1}\ \cdots \ {{\bf{x}}}_{p}]={[{\widetilde{{\bf{x}}}}_{1}\cdots {\widetilde{{\bf{x}}}}_{N}]}^{T}$ be the *N* × (*p* + 1) design matrix.

We consider horizontally partitioned data from *K* institutions or sites, all of which have the same set of features recorded for all of their subjects. **y** and *X*, known as the “pooled” outcome vector and the “pooled” design matrix respectively, can be decomposed by sites as follows:

$${\bf{y}}=\left(\begin{array}{l}{{\bf{y}}}^{(1)}\\ \vdots \\ {{\bf{y}}}^{(K)}\end{array}\right),\quad {\bf{X}}=\left(\begin{array}{l}{{\bf{X}}}^{(1)}\\ \vdots \\ {{\bf{X}}}^{(K)}\end{array}\right),$$

where **y**^(*k*)^ and **X**^(*k*)^ are the data from the *k*th site with *n*^(*k*)^ subjects, **X**^(*k*)^ is an *n*^(*k*)^ × (*p* + 1) matrix, and $N=\mathop{\sum }\nolimits_{k = 1}^{K}{n}^{(k)}$.

We first consider a univariate missing pattern where only *X*_1_ has missing values and the other variables are fully observed where *N*_*c*_ denotes the number of complete cases. We then consider general missing data patterns.

### Multiple imputation

An MI method replaces each missing value multiple times from its predictive distribution based on the observed data, accounting for the uncertainty of imputation. Each of the imputed datasets is analyzed separately as if it were fully observed. The results across all imputed datasets are then combined following Rubin’s rule. For example, if *X*_1_ which has missing values is continuous, we can use a Bayesian linear regression model for imputation

2 $${X}_{1}={\alpha }_{0}+{\alpha }_{1}Y+\mathop{\sum }\limits_{j=2}^{p}{\alpha }_{j}{X}_{j}+\zeta ,$$

where $\zeta \sim {\mathcal{N}}(0,{\tau }^{2})$ with priors

$$\pi ({\tau }^{2})\propto {\mathcal{IG}}(1/2,1/2),\quad {\boldsymbol{\alpha }}| {\tau }^{2} \sim {\mathcal{N}}({\bf{0}},{\tau }^{2}{\lambda }^{-1}{\bf{I}}),$$

where ${\mathcal{IG}}$ and ${\mathcal{N}}$ refer to the inverse gamma distribution and the multivariate Gaussian distribution, respectively. Let **Z** = [**1**, **y**, **x**_2_, …, **x**_*p*_], and let **Z**_*c*_ be the *N*_*c*_ × (*p* + 1) submatrix of **Z** loaded with the complete cases only. Similarly, let **x**_1,*c*_ be the subvector of **x**_1_ with the complete cases. The posterior distribution of (*τ*^2^, ***α***) is given by

3 $${\tau }^{2}| {{\bf{Z}}}_{c} \sim {\mathcal{IG}}(({N}_{c}+1)/2,(\rm{SSE}+1)/2),\\ {\boldsymbol{\alpha }}| {\tau }^{2},{{\bf{Z}}}_{c}\sim {\mathcal{N}}({({{\bf{Z}}}_{c}^{T}{{\bf{Z}}}_{c}+\lambda {\bf{I}})}^{-1}{{\bf{Z}}}_{c}^{T}{{\bf{x}}}_{1,c},{\tau }^{2}{({{\bf{Z}}}_{c}^{T}{{\bf{Z}}}_{c}+\lambda {\bf{I}})}^{-1}),$$

where ${\rm{SSE}}={{\bf{x}}}_{1,c}^{T}{{\bf{x}}}_{1,c}-{{\bf{x}}}_{1,c}^{T}{{\bf{Z}}}_{c}{({{\bf{Z}}}_{c}^{T}{{\bf{Z}}}_{c}+\lambda {\bf{I}})}^{-1}{{\bf{Z}}}_{c}^{T}{{\bf{x}}}_{1,c}$. The MI method samples (*τ*^2^, ***α***) from Equation (3), imputes the missing values of *X*_1_ according to Eq. (2) with random errors added, and fits the analysis model (1) using the imputed full data. This procedure is repeated multiple times.

When *X*_1_ that has missing values is binary, we can use a Bayesian logistic regression model for imputation with prior ${\boldsymbol{\alpha }} \sim {\mathcal{N}}\left({\bf{0}},{\lambda }^{-1}{\bf{I}}\right)$. Let $\widehat{{\boldsymbol{\alpha }}}$ be the maximum A posteriori estimator. Note that, as *N*_*c*_ tends to infinity, we have ${\rm{Cov}}(\widehat{{\boldsymbol{\alpha }}})={({{\bf{Z}}}_{c}^{T}{{\bf{W}}}_{c}{{\bf{Z}}}_{c}+\lambda {\bf{I}})}^{-1}(1+O({N}_{c}^{-1}))$, where **W** is a diagonal matrix with ${w}_{ii}={\rm{expit}}({\widetilde{{\bf{z}}}}_{i}^{T}\widehat{{\boldsymbol{\alpha }}})(1-{\rm{expit}}({\widetilde{{\bf{z}}}}_{i}^{T}\widehat{{\boldsymbol{\alpha }}}))$, **W**_*c*_ is the sub-diagonal-matrix of **W** for the complete cases, and ${\rm{expit}}(x)={{\rm{logit}}}^{-1}(x)=\frac{1}{1+{e}^{-x}}$. Therefore, the MI method samples ***α*** from ${\mathcal{N}}(\widehat{{\boldsymbol{\alpha }}},{({{\bf{Z}}}_{c}^{T}{{\bf{W}}}_{c}{{\bf{Z}}}_{c}+\lambda {\bf{I}})}^{-1})$, imputes the missing values according to the Bernoulli distribution, ${x}_{i1} \sim {\mathcal{B}}(1,{\rm{expit}}({\widetilde{{\bf{z}}}}_{i}^{T}{\boldsymbol{\alpha }}))$, and fits the analysis model using the imputed full data. This procedure is repeated multiple times. A regularization parameter *λ* can be used to avoid numerical difficulties particularly when the sample size at a site is less than the dimension of the parameters. We choose the value of *λ* to be as small as possible so that the bias caused by regularization can be negligible.

### Communication-efficient inference for distributed CD

One straightforward approach to analyze distributed data is to transmit the minimally sufficient information from all sites to the central site such that it would enable reproducing the results from analyzing data pooled from all sites. We call this approach the SI (suffcient information) method which will be extended to the sufficient information MI (siMI) in the next section. In linear regression, for example, we only need **X**^(*k*)*T*^**X**^(*k*)^ and **X**^(*k*)*T*^**y**^(*k*)^ to obtain the same least-square estimates for the regression coefficients as if we had the data pooled from all sites. This can be seen from the following equation:

4 $$\widehat{{\boldsymbol{\theta }}}={\left({{\bf{X}}}^{T}{\bf{X}}\right)}^{-1}\left({{\bf{X}}}^{T}{\bf{y}}\right)={\left(\sum _{k}{{\bf{X}}}^{(k)T}{{\bf{X}}}^{(k)}\right)}^{-1}\left(\sum _{k}{{\bf{X}}}^{(k)T}{{\bf{y}}}^{(k)}\right).$$

In addition, this approach requires only one single communication between each site and a central server where the computations described in Eq. (4) are conducted. To extend this strategy to the generalized linear models (GLMs), we note that a standard algorithm for fitting GLMs goes through Newton iterations, each of which requires the derivative and the curvature information of the global loglikelihood that involves regression coefficients. For example, in the logistic regression, the parameters are updated as follows for each iteration.

5 $${{\boldsymbol{\theta }}}^{(t+1)}\leftarrow {{\boldsymbol{\theta }}}^{(t)}+s{\left(\sum _{k}{{\bf{X}}}^{(k)T}{{\bf{W}}}^{(k)}{{\bf{X}}}^{(k)}\right)}^{-1}\left(\sum _{k}{{\bf{X}}}^{(k)T}({{\bf{y}}}^{(k)}-{{\boldsymbol{\pi }}}^{(k)})\right),$$

where ${\pi }_{i}^{(k)}={\rm{expit}}({\widetilde{{\bf{x}}}}_{i}^{(k)T}{{\boldsymbol{\theta }}}^{(t)})$, **W**^(*k*)^ = diag(***π***^(*k*)^)diag(**1** − ***π***^(*k*)^), and *s* is the step size. For each iteration, the central site has to transfer ***θ***^(*t*)^ to other sites and all sites have to transfer **X**^(*k*)*T*^**W**^(*k*)^**X**^(*k*)^ and **X**^(*k*)*T*^(**y**^(*k*)^ − ***π***^(*k*)^) back to the central site. Since the number of required round-trip communications is same as that of Newton iterations, the SI method may not be communication efficient. This approach is privacy preserving in the sense that the subject-level data are not shared outside of each site and hence are protected. Again, this approach generates the same results as the analysis of the pooled data.

We also consider two alternative distributed analysis methods that are communication efficient^22^ proposed the average mixture algorithm. Each site estimates the model parameters using the data available at the site only, and then combine the estimates to find the global estimate for the parameters. Let ${\widehat{{\boldsymbol{\theta }}}}^{(k)}$ be the estimate from the *k*th site. Then, we have

6 $${\widehat{{\boldsymbol{\theta }}}}_{avgm}=\sum _{k}{w}_{k}{\widehat{{\boldsymbol{\theta }}}}^{(k)},$$

where *w*_*k*_ ≥ 0 and ∑_*k*_*w*_*k*_ = 1. Note that the weights *w*_*k*_ should reflect the sample size of each site. This method is communication efficient as it requires a single one-way communication only. The resulting estimator can achieve the best rate of convergence in asymptotics^22^. However, the local estimates can be volatile, especially when the sample size of the site is small. Therefore, the finite sample characteristic of ${\widehat{{\boldsymbol{\theta }}}}_{avgm}$ can be quite different. We call this approach the AVGM (average mixture) algorithm. Jordan et al.^23^ proposed using the curvature information from one central site, say site 1, and the global derivative at a point near the true parameter. The estimator is defined as the minimizer of the CSL, which is defined as

$$\widetilde{{\mathcal{L}}}({\boldsymbol{\theta }})={{\mathcal{L}}}_{1}({\boldsymbol{\theta }})-\langle \nabla {{\mathcal{L}}}_{1}(\overline{{\boldsymbol{\theta }}})-\nabla {\mathcal{L}}(\overline{{\boldsymbol{\theta }}}),{\boldsymbol{\theta }}\rangle ,$$

where ${\mathcal{L}}$ is the gross average loglikelihood (loss function), ${{\mathcal{L}}}_{1}$ is the local average loglikelihood at the central site (site 1), $\overline{{\boldsymbol{\theta }}}$ is a point close to the true ***θ***, and 〈⋅,⋅〉 denotes the inner product. Note that it requires one round-trip communication in order to calculate $\nabla {\mathcal{L}}(\overline{{\boldsymbol{\theta }}})$. The solution achieves the optimal convergence rate if $\overline{{\boldsymbol{\theta }}}$ converges fast enough^23^. However, the finite sample performance may deteriorate if the sample size at the central site is small, as it utilizes the curvature information from the central site only and the initial solution $\overline{{\boldsymbol{\theta }}}$ is also obtained from the central site only. We call this approach the CSL method.

 Jordan et al.^23^ proposes multiple versions of CSL methods including the ones that repeat the whole procedure using the current solution as a new initial coefficient $\overline{{\boldsymbol{\theta }}}$. Since those approaches require more communications, we do not consider all of them and restrict our focus on the most communication-efficient version, the one described above. The two communication-efficient methods AVGM and CSL are also privacy-preserving in the sense that the individual level data are not shared between sites.

### Distributed MI for univariate missing data pattern

One straightforward way to impute missing values for distributed data is to impute missing data in each site separately using a standard MI method. We call this approach the iMI method. When using iMI, no subject-level data are shared across sites and thus no communication is required. As such, it is communication-efficient and privacy-preserving. However, this approach has a number of limitations as discussed in the “Introduction” section.

#### Algorithm 1

Independent MI algorithm

**Table:** 

1	**for** *k* ← 1 **to** *K* **do**
2	Fit the imputation model at site *k* to find ${\widehat{{\boldsymbol{\alpha }}}}^{(k)}$ and ${\rm{Cov}}({\widehat{{\boldsymbol{\alpha }}}}^{(k)})$;
3	Sample ${{\boldsymbol{\alpha }}}_{1}^{(k)},\ldots ,{{\boldsymbol{\alpha }}}_{M}^{(k)}$ independently from ${\mathcal{N}}({\widehat{{\boldsymbol{\alpha }}}}^{(k)},{\rm{Cov}}({\widehat{{\boldsymbol{\alpha }}}}^{(k)}))$;
4	**end**
5	**for** *m* ← 1 **to** *M* **do**
6	**for** *k* ← 1 **to** *K* **do** Impute the missing data at site *k* based on ${{\boldsymbol{\alpha }}}_{m}^{(k)}$
7	Fit the analysis model and obtain ${\widehat{{\boldsymbol{\theta }}}}_{m}$ and ${\rm{Cov}}({\widehat{{\boldsymbol{\theta }}}}_{m})$;
8	**end**
9	Combine the results by Rubin’s rule to obtain $\widehat{{\boldsymbol{\theta }}}$ and ${\rm{Cov}}(\widehat{{\boldsymbol{\theta }}})$;

An alternative approach is to use the SI method to fit a distributed imputation model, which would be equivalent to fit the imputation model using data pooled from all sites. The imputation approach requires as many communications as the number of communications required by the SI method plus a one-way communication to deliver the sampled imputation model parameters to all sites. Hence, this method is less communication-efficient than the other methods considered, unless the imputation model is a linear regression. However, as it uses the full information, it is expected to yield the best imputation performance. We call this approach the siMI method, which will be used as a benchmark for assessing imputation performance in our numerical studies.

#### Algorithm 2

Sufficient information MI algorithm

**Table:** 

1	Fit the global imputation model using the SI method to find ${\widehat{{\boldsymbol{\alpha }}}}_{si}$ and ${\rm{Cov}}({\widehat{{\boldsymbol{\alpha }}}}_{si})$;
2	Sample ***α***_1_, …, ***α***_*M*_ independently from ${\mathcal{N}}({\widehat{{\boldsymbol{\alpha }}}}_{si},{\rm{Cov}}({\widehat{{\boldsymbol{\alpha }}}}_{si}))$;
3	Send ***α***_1_, …, ***α***_*M*_ to all sites;
4	**for** *m* ← 1 **to** *M* **do**
5	**for** *k* ← 1 **to** *K* **do** Impute the missing data at site *k* based on ***α***_*m*_;
6	Fit the analysis model and obtain ${\widehat{{\boldsymbol{\theta }}}}_{m}$ and ${\rm{Cov}}({\widehat{{\boldsymbol{\theta }}}}_{m})$;
7	**end**
8	Combine the results by Rubin’s rule to obtain $\widehat{{\boldsymbol{\theta }}}$ and ${\rm{Cov}}(\widehat{{\boldsymbol{\theta }}})$;

We also develop two communication-efficient distributed MI methods, namely, avgmMI and cslMI, which adapt the communication-efficient methods described above. The avgmMI method fits the distributed imputation model using the AVGM method, in which the weights are chosen to be proportional to the number of complete cases in each site.

$${\widehat{{\boldsymbol{\alpha }}}}_{avgm}=\frac{1}{{N}_{c}}\sum _{k}{n}_{c}^{(k)}{\widehat{{\boldsymbol{\alpha }}}}^{(k)},$$

where the covariance matrix of ${\widehat{{\boldsymbol{\alpha }}}}_{avgm}$ is given by

$${\rm{Cov}}({\widehat{{\boldsymbol{\alpha }}}}_{avgm})=\frac{1}{{N}_{c}^{2}}\sum _{k}{n}_{c}^{(k)2}{\rm{Cov}}({\widehat{{\boldsymbol{\alpha }}}}^{(k)}).$$

Since each site needs to transmit two quantities ${\widehat{{\boldsymbol{\alpha }}}}^{(k)}$ and ${\rm{Cov}}({\widehat{{\boldsymbol{\alpha }}}}^{(k)})$ to the central site, avgmMI requires two one-way communications. This is a huge advantage over the siMI method in terms of communication cost except when the imputation model is a linear regression.

#### Algorithm 3

AVGM MI algorithm

**Table:** 

1	**for** *k* ← 1 **to** *K* **do**
2	Find the estimates ${\widehat{{\boldsymbol{\alpha }}}}^{(k)}$ and ${\rm{Cov}}({\widehat{{\boldsymbol{\alpha }}}}^{(k)})$ at site *k*;
3	Send ${\widehat{{\boldsymbol{\alpha }}}}^{(k)}$ and ${\rm{Cov}}({\widehat{{\boldsymbol{\alpha }}}}^{(k)})$ to the central site;
4	**end**
5	${\widehat{{\boldsymbol{\alpha }}}}_{avgm}\leftarrow \frac{1}{{N}_{c}}{\sum }_{k}{n}_{c}^{(k)}{\widehat{{\boldsymbol{\alpha }}}}^{(k)}$;
6	${\rm{Cov}}({\widehat{{\boldsymbol{\alpha }}}}_{avgm})\leftarrow \frac{1}{{N}_{c}^{2}}{\sum }_{k}{n}_{c}^{(k)2}{\rm{Cov}}({\widehat{{\boldsymbol{\alpha }}}}^{(k)})$;
7	Sample ***α***_1_, …, ***α***_*M*_ independently from ${\mathcal{N}}({\widehat{{\boldsymbol{\alpha }}}}_{avgm},{\rm{Cov}}({\widehat{{\boldsymbol{\alpha }}}}_{avgm}))$;
8	Send ***α***_1_, …, ***α***_*M*_ to all sites;
9	**for** *m* ← 1 **to** *M* **do**
10	**for** *k* ← 1 **to** *K* **do** Impute the missing data at site *k* based on ***α***_*m*_
11	Fit the analysis model and obtain ${\widehat{{\boldsymbol{\theta }}}}_{m}$ and ${\rm{Cov}}({\widehat{{\boldsymbol{\theta }}}}_{m})$;
12	**end**
13	Combine the results by Rubin’s rule to obtain $\widehat{{\boldsymbol{\theta }}}$ and ${\rm{Cov}}(\widehat{{\boldsymbol{\theta }}})$;

The cslMI method fits the imputation model using the CSL method.

$${\widehat{{\boldsymbol{\alpha }}}}_{csl}={\arg \min }_{{\boldsymbol{\alpha }}}\widetilde{{\mathcal{L}}}({\boldsymbol{\alpha }}),$$

where $\overline{{\boldsymbol{\alpha }}}$ is chosen as the local solution at the central site.

$$\overline{{\boldsymbol{\alpha }}}={\arg \min }_{{\boldsymbol{\alpha }}}{{\mathcal{L}}}_{1}({\boldsymbol{\alpha }}).$$

Following the asymptotic property of the CSL estimator^23^, the covariance matrix of ${\widehat{{\boldsymbol{\alpha }}}}_{csl}$ is consistently estimated by

$${\rm{Cov}}({\widehat{{\boldsymbol{\alpha }}}}_{csl})=\frac{1}{{N}_{c}}{\nabla }^{2}{{\mathcal{L}}}_{1}{({\boldsymbol{\alpha }})}^{-1}{| }_{{\boldsymbol{\alpha }} = {\widehat{{\boldsymbol{\alpha }}}}_{csl}}.$$

#### Algorithm 4

CSL MI algorithm

**Table:** 

1	Find the estimate $\overline{{\boldsymbol{\alpha }}}={\arg \min }_{{\boldsymbol{\alpha }}}{{\mathcal{L}}}_{1}({\boldsymbol{\alpha }})$, which is the optimal estimate at site 1;
2	Send $\overline{{\boldsymbol{\alpha }}}$ to all sites and receive $\nabla {{\mathcal{L}}}_{k}({\boldsymbol{\alpha }}){| }_{{\boldsymbol{\alpha }} = \overline{{\boldsymbol{\alpha }}}}$ back;
3	Find ${\widehat{{\boldsymbol{\alpha }}}}_{csl}={\arg \min }_{{\boldsymbol{\alpha }}}\widetilde{{\mathcal{L}}}({\boldsymbol{\alpha }})$ and ${\rm{Cov}}({\widehat{{\boldsymbol{\alpha }}}}_{csl})=\frac{1}{{N}_{c}}{\nabla }^{2}{{\mathcal{L}}}_{1}{({\boldsymbol{\alpha }})}^{-1}{| }_{{\boldsymbol{\alpha }} = {\widehat{{\boldsymbol{\alpha }}}}_{csl}}$;
4	Sample ***α***_1_, …, ***α***_*M*_ independently from ${\mathcal{N}}({\widehat{{\boldsymbol{\alpha }}}}_{csl},{\rm{Cov}}({\widehat{{\boldsymbol{\alpha }}}}_{csl}))$;
5	Send ***α***_1_, …, ***α***_*M*_ to all sites;
6	**for** *m* ← 1 **to** *M* **do**
7	**for** *k* ← 1 **to** *K* **do** Impute the missing data at site *k* based on ***α***_*m*_;
8	Fit the analysis model and obtain ${\widehat{{\boldsymbol{\theta }}}}_{m}$ and ${\rm{Cov}}({\widehat{{\boldsymbol{\theta }}}}_{m})$;
9	**end**
10	Combine the results by Rubin’s rule to obtain $\widehat{{\boldsymbol{\theta }}}$ and ${\rm{Cov}}(\widehat{{\boldsymbol{\theta }}})$;

The aforementioned algorithms can be used for a wide range of imputation models including, but not limited to, GLMs. But, when applied to a linear imputation model, the algorithms need to be designed to draw the error variance parameter *τ*^2^ in different ways. The iMI and siMI methods can follow the procedure in (3) without modification. However, a direct application of the AVGM and CSL approaches would sample the error variance *τ*^2^ from Gaussian, which does not ensure its positiveness. To address this issue, we propose the following alternative procedures for sampling *τ*^2^.

For avgmMI, we use SSE = ∑_*k*_SSE^(*k*)^ where SSE^(*k*)^ is the sum of squared errors from site *k*.

$${\rm{SSE}}^{(k)}=\, \parallel {{\bf{x}}}_{1,c}^{(k)}-{{\bf{Z}}}_{c}^{(k)}{\widehat{{\boldsymbol{\alpha }}}}^{(k)}{\parallel }_{2}^{2}.$$

The error variance is sampled from ${\tau }^{2} \sim {\mathcal{IG}}({N}_{c}/2,{\rm{SSE}}/2)$ and ***α*** is sampled from the multivariate Gaussian distribution with mean ${\widehat{{\boldsymbol{\alpha }}}}_{avgm}$ and the variance

$${\rm{Cov}}({\widehat{{\boldsymbol{\alpha }}}}_{avgm}| {\tau }^{2})=\frac{{\tau }^{2}}{{N}_{c}^{2}}\sum _{k}{n}_{c}^{(k)2}{({{\bf{Z}}}_{c}^{(k)T}{{\bf{Z}}}_{c}^{(k)}+\lambda {\bf{I}})}^{-1}.$$

Each site needs transfer ${\widehat{{\boldsymbol{\alpha }}}}^{(k)}$, SSE^(*k*)^, and ${({{\bf{Z}}}_{c}^{(k)T}{{\bf{Z}}}_{c}^{(k)}+\lambda {\bf{I}})}^{-1}$ to the central site after fitting the local imputation model.

The cslMI method also follows the procedure in (3). It entails sampling *τ*^2^ with ${\rm{SSE}} = N_c \parallel {{\bf{x}}}_{1,c}^{(1)}-{{\bf{Z}}}_{c}^{(1)}{\widehat{{\boldsymbol{\alpha }}}}_{csl}{\parallel }_{2}^{2}/{n}_{c}^{(1)}$, which is based on the asymptotic variance of $\widehat{{\boldsymbol{\alpha }}}$ defined in Eq. (13) in ref. ^23^ and does not require additional communication between sites to compute, and then sampling ***α*** from the multivariate Gaussian distribution with mean ${\widehat{{\boldsymbol{\alpha }}}}_{csl}$ and the variance

$${\rm{Cov}}({\widehat{{\boldsymbol{\alpha }}}}_{csl}| {\tau }^{2})=\frac{{\tau }^{2}{n}_{c}^{(1)}}{{N}_{c}}{({{\bf{Z}}}_{c}^{(1)T}{{\bf{Z}}}_{c}^{(1)}+\lambda {\bf{I}})}^{-1}.$$

In finite samples, the above-mentioned asymptotic approximation of SSE may contribute to deteriorating performance of the cslMI when sample size decreases, as evidenced in our numerical experiments.

### Distributed MI for general missing data patterns

Since it is commonly encountered in practice to have multiple variables with missing values, it is of particular interest to develop privacy-preserving distributed MI methods for general missing data patterns.

A very popular method for handling general missing data patterns is the MI by chained equation, known as MICE in short^20^. Without loss of generality, we assume that the first *q* (*q* < *p*) covariates, i.e., (*X*_1_, . . . , *X*_*q*_), have missing values. The MICE algorithm starts with an initial imputation. For example, the missing values of *X*_*j*_ can be imputed by random samples from the observed data. Then, for each *j* = 1, …, *q*, the missing values of *X*_*j*_ are imputed by a MI method, assuming that the imputed values of all the other variables were actually observed. A sweep of imputations for all *q* missing variables form an iteration, and multiple iterations are fulfilled until the distribution of imputed values becomes stationary before the first imputed dataset is sampled. Multiple iterations are carried out between each imputed dataset to alleviate autocorrelations. A total of *M* imputed datasets are collected and used to fit the analysis model separately, and the results are combined by the Rubin’s rule. Readers are referred to ref. ^20^ for more details about MICE.

#### Algorithm 5

Privacy-preserving MI by chained equation algorithm

**Table:** 

1	**for** *j* ← 1 **to** *q* **do**
2	Impute the missing values of *X*_*j*_.
3	**end**
4	**for** *m* ← 1 **to** *M* **do**
5	**repeat**
6	**for** *j* ← 1 **to** *q* **do**
7	Fit the imputation model for *X*_*j*_ using the samples for which *X*_*j*_ are observed and impute the missing values of *X*_*j*_ by iMI, avgmMI, cslMI, or siMI;
8	**end**
9	**until** multiple times;
10	Fit the analysis model and obtain ${\widehat{{\boldsymbol{\theta }}}}_{m}$ and ${\rm{Cov}}({\widehat{{\boldsymbol{\theta }}}}_{m})$;
11	**end**
12	Combine the results by Rubin’s rule to obtain $\widehat{{\boldsymbol{\theta }}}$ and ${\rm{Cov}}(\widehat{{\boldsymbol{\theta }}})$;

In parallel with the distributed MI methods, we consider four privacy-preserving distributed MICE approaches, namely, iMICE, avgmMICE, cslMICE, and siMICE. The generic privacy-preserving MICE algorithm is summarized in Box 5. Unlike the distributed MI methods for the univariate missing pattern, each imputation model is fitted multiple times in MICE. Therefore, these distributed MICE methods may not be communication-efficient except iMICE, which requires no communication between each site and the central site.

### Reporting summary

Further information on research design is available in the Nature Research Reporting Summary linked to this article.

## Supplementary information

Reporting Summary

## Data Availability

The real data analyzed in this article were provided by the Georgia Coverdell Acute Stroke Registry and restrictions apply to the availability of these data. Request for access to the data should be submitted to and approved by the Georgia Coverdell Acute Stroke Registry.

## Code availability

We used R software (Version 3.6.3) including custom code and existing R package (mice) to conduct the simulations and real data analysis. All relevant R codes are publicly available from https://github.com/changgee/MIDist.
