# Supplementary material for: Multiple imputation for analysis of incomplete data in distributed health data networks
Source: Nat Commun. 2020 Oct 29;11:5467. doi: 10.1038/s41467-020-19270-2 (PMC7596726; doi:10.1038/s41467-020-19270-2)
Supplement: Supplementary file 1 — Reporting Summary [file 41467_2020_19270_MOESM1_ESM.pdf]

nature research

Corresponding author(s): Qi Long

Last updated by author(s): Sep 28, 2020

Reporting Summary

Nature Research wishes to improve the reproducibility of the work that we publish. This form provides structure for consistency and transparency in reporting. For further information on Nature Research policies, see our Editorial Policies and the Editorial Policy Checklist.

Statistics

For all statistical analyses, confirm that the following items are present in the figure legend, table legend, main text, or Methods section.

n/a	Confirmed	
☐	☒	The exact sample size (n) for each experimental group/condition, given as a discrete number and unit of measurement
☐	☒	A statement on whether measurements were taken from distinct samples or whether the same sample was measured repeatedly
☐	☒	The statistical test(s) used AND whether they are one- or two-sided<br>*Only common tests should be described solely by name; describe more complex techniques in the Methods section.*
☐	☒	A description of all covariates tested
☐	☒	A description of any assumptions or corrections, such as tests of normality and adjustment for multiple comparisons
☐	☒	A full description of the statistical parameters including central tendency (e.g. means) or other basic estimates (e.g. regression coefficient) AND variation (e.g. standard deviation) or associated estimates of uncertainty (e.g. confidence intervals)
☐	☒	For null hypothesis testing, the test statistic (e.g. F, t, r) with confidence intervals, effect sizes, degrees of freedom and P value noted<br>*Give P values as exact values whenever suitable.*
☒	☐	For Bayesian analysis, information on the choice of priors and Markov chain Monte Carlo settings
☒	☐	For hierarchical and complex designs, identification of the appropriate level for tests and full reporting of outcomes
☒	☐	Estimates of effect sizes (e.g. Cohen's d, Pearson's r), indicating how they were calculated

Our web collection on statistics for biologists contains articles on many of the points above.

Software and code

Policy information about availability of computer code

Data collection	We analyze the de-identified real data from the Georgia Coverdell Acute Stroke Registry (GCASR) . Our work did not involve new data collection.
Data analysis	We used R software (Version 3.6.3) including our custom code and the mice package to conduct simulations and real data analysis. The relevant R code for implementing the proposed methods and for the simulation is publicly available on GitHub (https://github.com/changgee/MIDist).

For manuscripts utilizing custom algorithms or software that are central to the research but not yet described in published literature, software must be made available to editors and reviewers. We strongly encourage code deposition in a community repository (e.g. GitHub). See the Nature Research guidelines for submitting code & software for further information.

Data

Policy information about availability of data

All manuscripts must include a data availability statement. This statement should provide the following information, where applicable:

- Accession codes, unique identifiers, or web links for publicly available datasets
- A list of figures that have associated raw data
- A description of any restrictions on data availability

In this article, we analyzed the de-identified real data from the Georgia Coverdell Acute Stroke Registry (GCASR) and restrictions apply to the availability of these data. Request for access to the data should be submitted to and approved by the Georgia Coverdell Acute Stroke Registry.

Field-specific reporting

Please select the one below that is the best fit for your research. If you are not sure, read the appropriate sections before making your selection.

- [x] Life sciences
- [ ] Behavioural & social sciences
- [ ] Ecological, evolutionary & environmental sciences

For a reference copy of the document with all sections, see nature.com/documents/nr-reporting-summary-flat.pdf

Life sciences study design

All studies must disclose on these points even when the disclosure is negative.

Sample size	The goal of this paper is to develop distributed multiple imputation (MI) methods. We evaluated the performance of our distributed MI methods in simulations and in analysis of the de-identified data from the Georgia Coverdell Acute Stroke Registry (GCASR). Our study did not involve new data collection. Sample size calculations are not applicable. The GCASR data set includes data from multiple hospitals which allow us to mimic a distributed health data network (DHDN) where health data are horizontally partitioned across hospitals and subject-level data cannot be shared or sent to a central data repository. As such, it is sufficient to demonstrate the performance of our proposed distributed MI methods.
Data exclusions	No data were excluded.
Replication	The goal of this paper is to develop distributed multiple imputation (MI) methods. The performance of our distributed MI methods was evaluated and replicated in both extensive simulation studies and in analysis of the de-identified real data from the Georgia Coverdell Acute Stroke Registry (GCASR).
Randomization	Our study did not involve conducting a prospective study or collection of new data, so randomization is not applicable.
Blinding	Our study did not involve conducting a prospective study or collection of new data, so blinding is not applicable.

Reporting for specific materials, systems and methods

We require information from authors about some types of materials, experimental systems and methods used in many studies. Here, indicate whether each material, system or method listed is relevant to your study. If you are not sure if a list item applies to your research, read the appropriate section before selecting a response.

Materials & experimental systems

n/a	Involved in the study	
x		Antibodies
x		Eukaryotic cell lines
x		Palaeontology and archaeology
x		Animals and other organisms
x		Human research participants
x		Clinical data
x		Dual use research of concern

Methods

n/a	Involved in the study	
x		ChIP-seq
x		Flow cytometry
x		MRI-based neuroimaging
